# Supplementary material for: Reliable Prediction of Caco-2 Permeability by Supervised Recursive Machine Learning Approaches
Source: Pharmaceutics. 2022 Sep 21;14(10):1998. doi: 10.3390/pharmaceutics14101998 (PMC9610902; doi:10.3390/pharmaceutics14101998)
Supplement: Supplementary file 1 [file pharmaceutics-14-01998-s001.zip › pharmaceutics-1899591-supplementary.pdf]

Reliable prediction of Caco-2 permeability by supervised recursive machine learning approaches

Gabriela Falcón-Cano [1], Christophe Molina [2], and Miguel Ángel Cabrera-Pérez [1 3]

[1] Unidad de Modelación y Experimentación Biofarmacéutica. Centro de Bioactivos Químicos. Universidad Central "Marta Abreu" de las Villas. Santa Clara 54830, Villa Clara, Cuba
[2] PIKAÏROS, S.A, 31650, Saint Orens de Gameville, France
[3] Departamento de Ciencias Farmacéuticas, Facultad de Ciencias, Universidad Católica del Norte, Angamos 0610, Antofagasta, Chile
[*] Correspondence: gfalcon@uclv.cu (GFC); christophe.molina@pikairos.com (CM); macabreraster@gmail.com (MACP)

Tables

Supplementary Table S1. Final list of most important variables sorted by number of occurrences

Descriptors		Number of occurrences
SlogP	Log of the octanol/water partition coefficient (including implicit hydrogens). Captures lipophilicity	8222
SMR	Molecular Refractivity (including implicit hydrogens). Captures polarizability and protonation state	1706
TPSA	Topological Polar Surface Area. Captures polarizability	1269
Hallkier alpha	Hall Kier Alpha value. Captures polarizability	549
Kappa 3	Molecular shape index. Captures flexibility	352

Figures

Supplementary Figure S1. Frequency distribution histograms for slogP, MW, HBD, HBA, RBN and TPSA

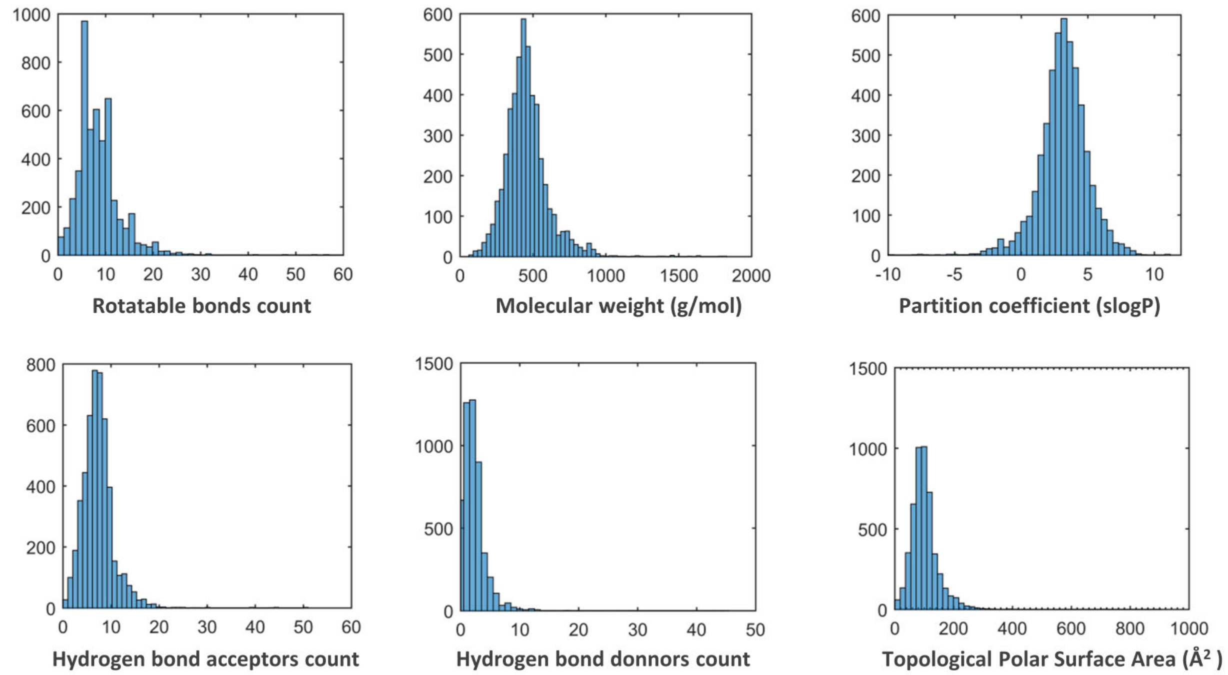

Supplementary Figure S2. Comparison of the molecular space of CLEANED and RELIABLE Sets.

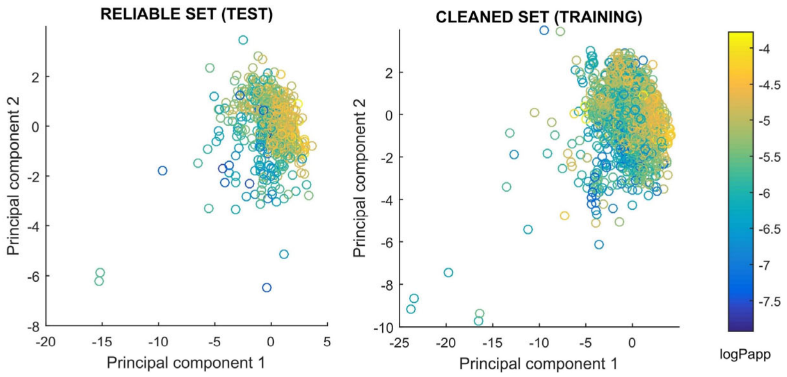

Inter- & Intra-set similarity distributions based on the nearest neighbors search

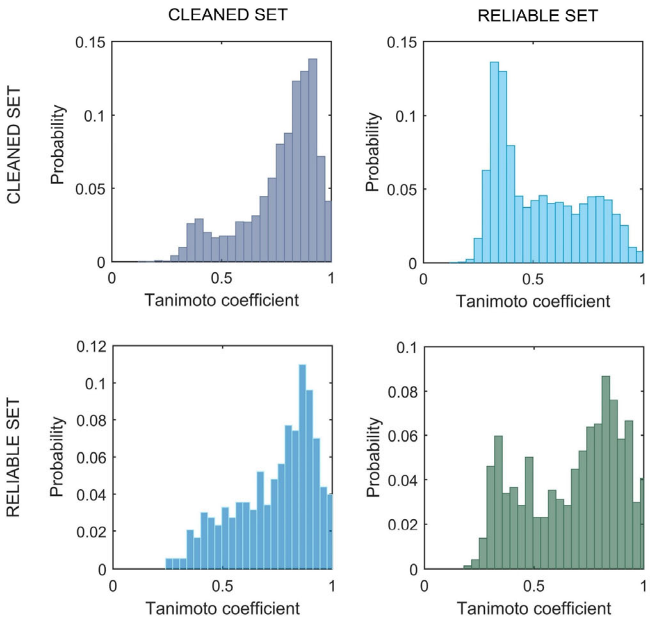

Supplementary Figure S3. Regression plots for External and Reliable Validation Sets.

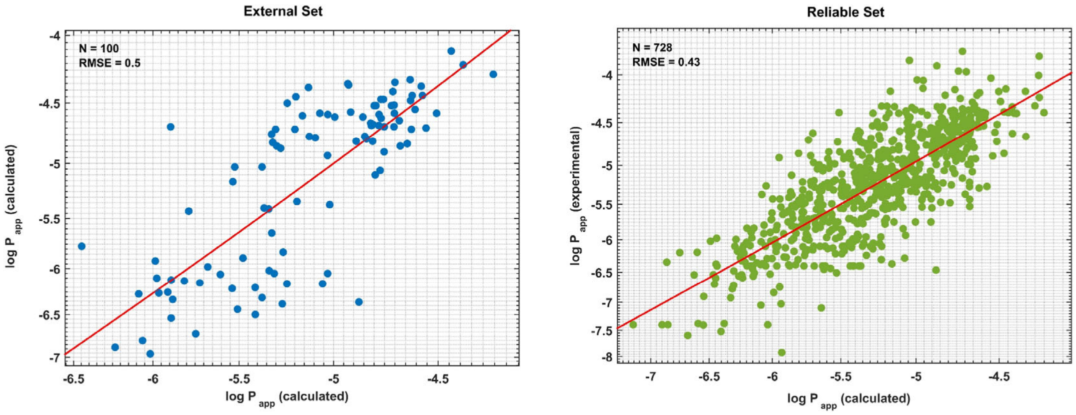

Supplementary Figure S4. Correlation matrix of 5-most important variables.

	slogP	*SMR*	*TPSA*	*Kappa 3*	*HallKier Alpha*
SlogP	1.00	0.32	-0.32	0.05	-0.22
SMR	0.32	1.00	0.68	0.69	-0.76
TPSA	-0.32	0.68	1.00	0.78	-0.65
Kappa 3	0.05	0.78	0.69	1.00	-0.54
HallKier Alpha	-0.22	-0.76	-0.65	-0.54	1.00

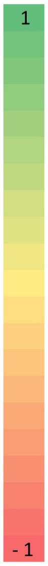

Pearson Correlation Coefficient
